# Supplementary figures and images for: Identification of resistance mechanisms to small-molecule inhibition of TEAD-regulated transcription
Source: EMBO Rep. 2024 Aug 5;25(9):14. doi: 10.1038/s44319-024-00217-3 (PMC11387499; doi:10.1038/s44319-024-00217-3)

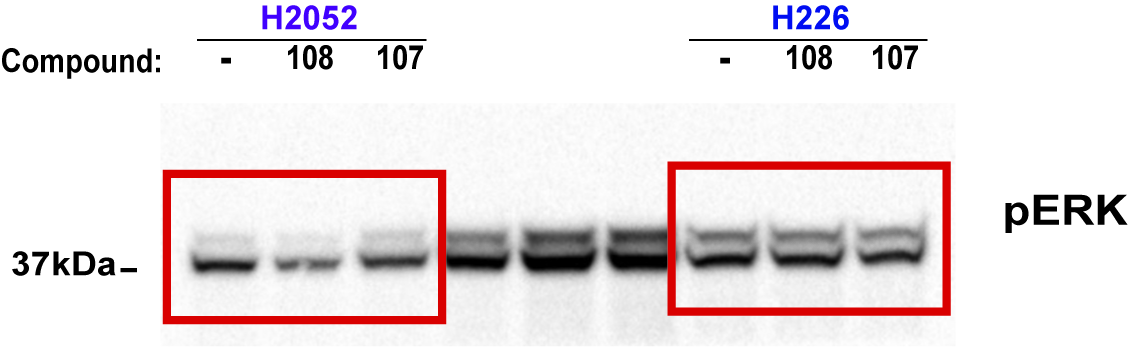

Supplement: Supplementary file 8 — Source data Fig. 1 [file 44319_2024_217_MOESM8_ESM.zip › Figure 1/1I/western pERK.tif]

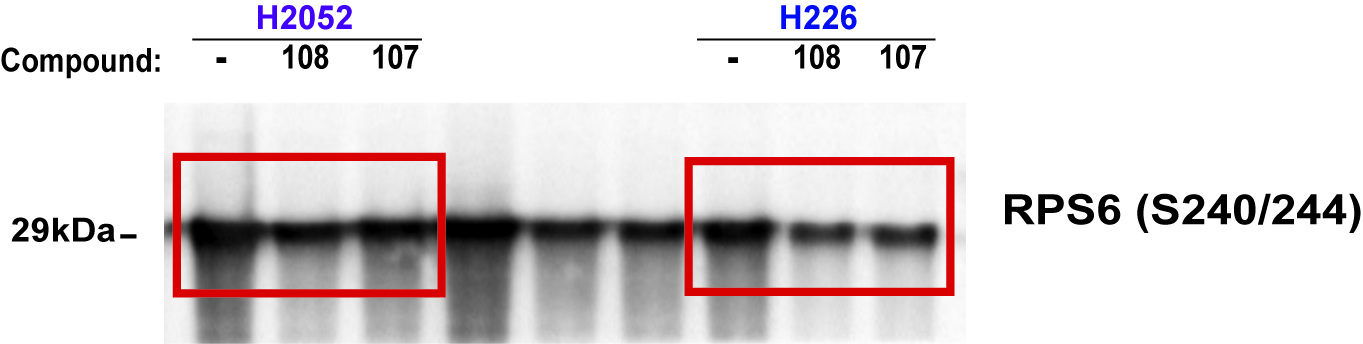

Supplement: Supplementary file 8 — Source data Fig. 1 [file 44319_2024_217_MOESM8_ESM.zip › Figure 1/1I/western RPS6 240_244.tif]

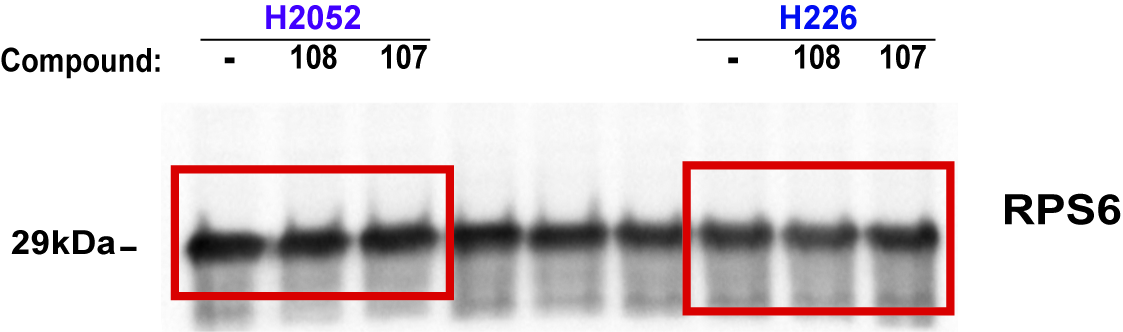

Supplement: Supplementary file 8 — Source data Fig. 1 [file 44319_2024_217_MOESM8_ESM.zip › Figure 1/1I/western RPS6.tif]

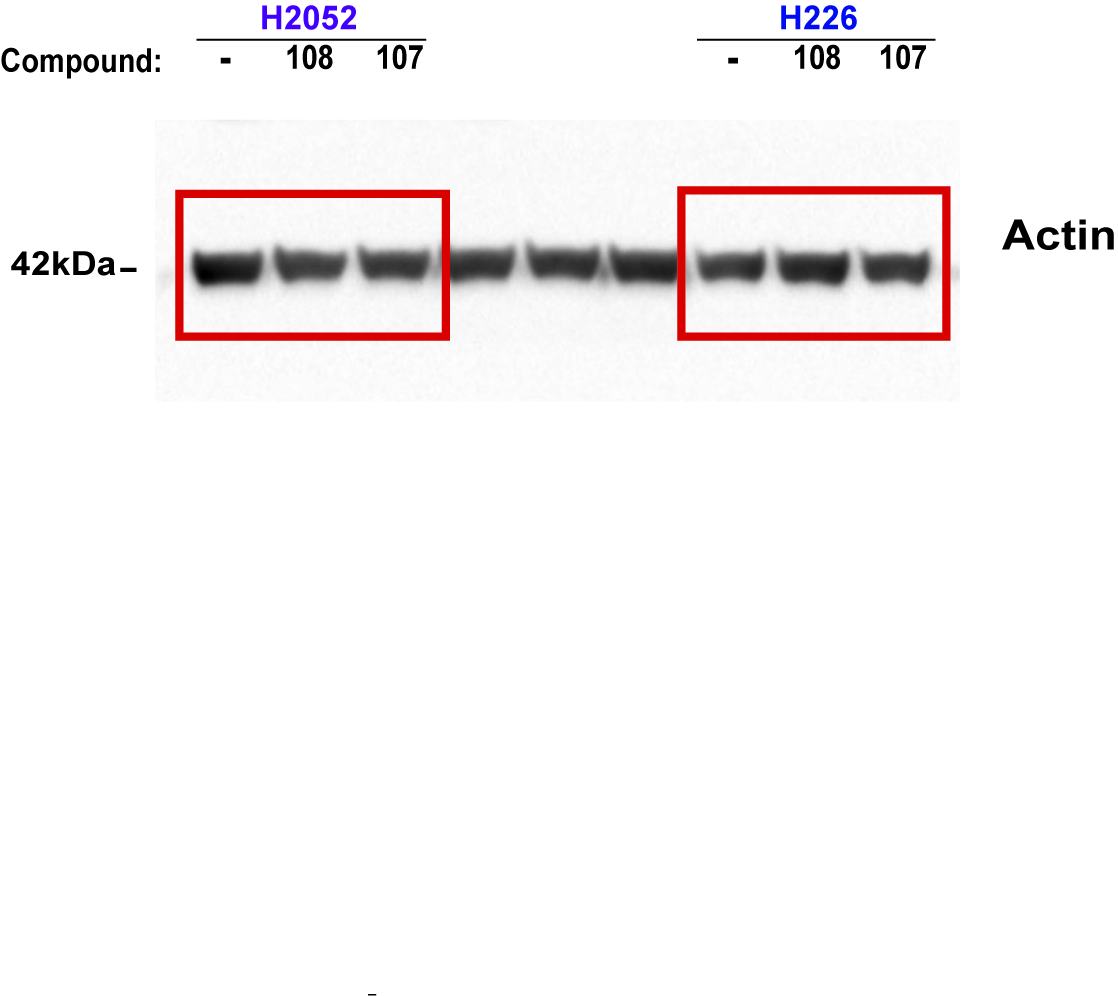

Supplement: Supplementary file 8 — Source data Fig. 1 [file 44319_2024_217_MOESM8_ESM.zip › Figure 1/1I/western actin.tif]

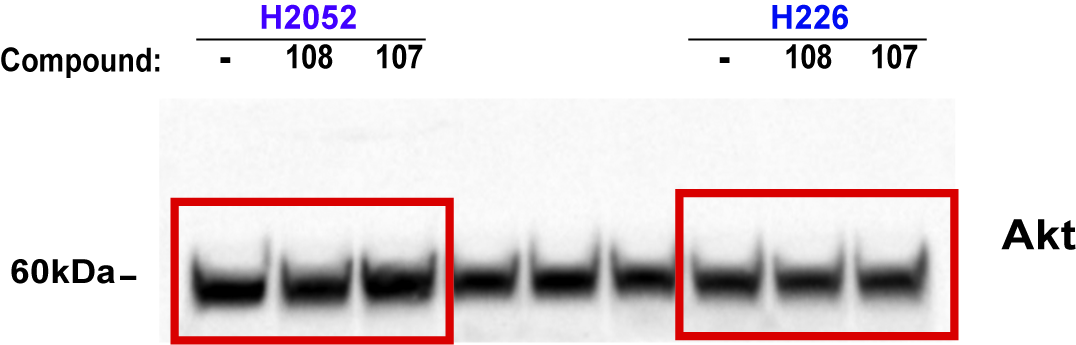

Supplement: Supplementary file 8 — Source data Fig. 1 [file 44319_2024_217_MOESM8_ESM.zip › Figure 1/1I/western Akt.tif]

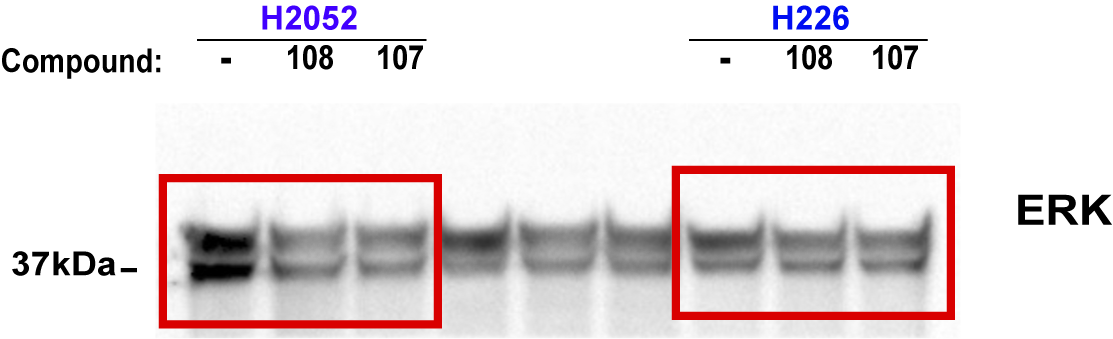

Supplement: Supplementary file 8 — Source data Fig. 1 [file 44319_2024_217_MOESM8_ESM.zip › Figure 1/1I/western ERK.tif]

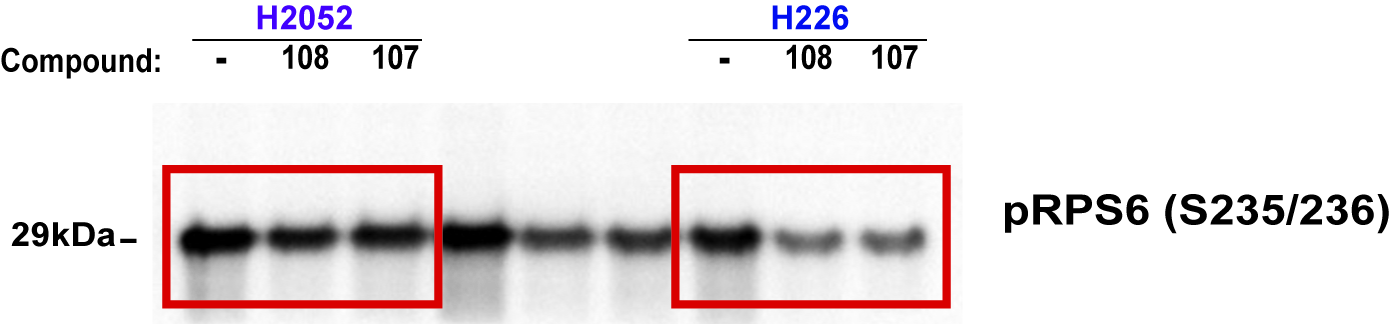

Supplement: Supplementary file 8 — Source data Fig. 1 [file 44319_2024_217_MOESM8_ESM.zip › Figure 1/1I/western RPS6 235_236.tif]

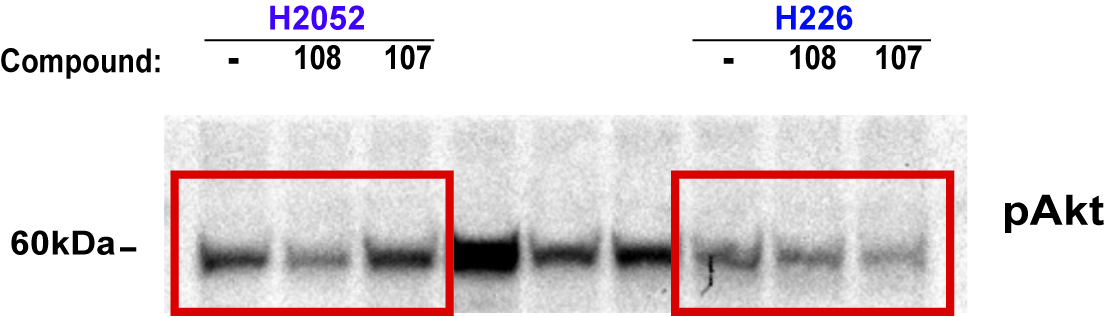

Supplement: Supplementary file 8 — Source data Fig. 1 [file 44319_2024_217_MOESM8_ESM.zip › Figure 1/1I/western pAkt.tif]

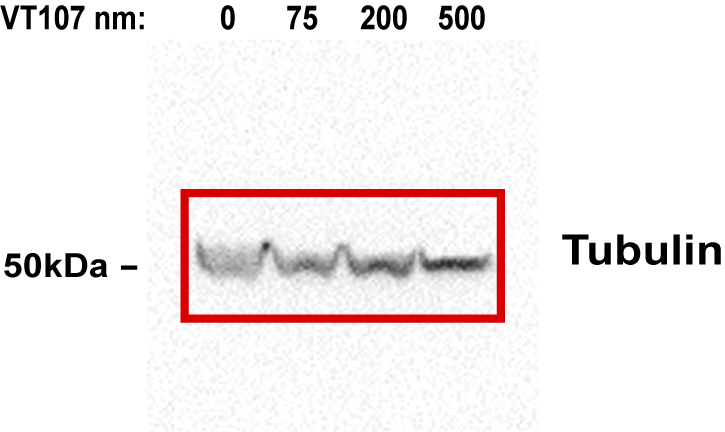

Supplement: Supplementary file 8 — Source data Fig. 1 [file 44319_2024_217_MOESM8_ESM.zip › Figure 1/1C/western Tubulin.tif]

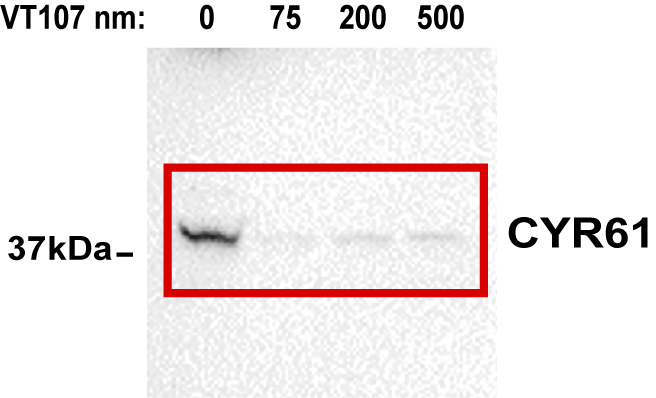

Supplement: Supplementary file 8 — Source data Fig. 1 [file 44319_2024_217_MOESM8_ESM.zip › Figure 1/1C/western CYR61.tif]

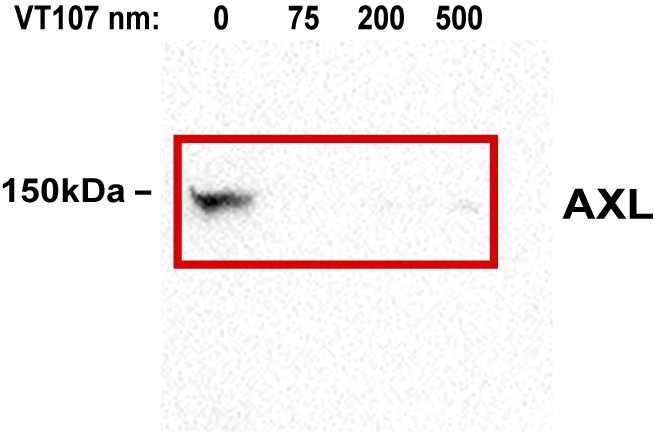

Supplement: Supplementary file 8 — Source data Fig. 1 [file 44319_2024_217_MOESM8_ESM.zip › Figure 1/1C/western AXL.tif]

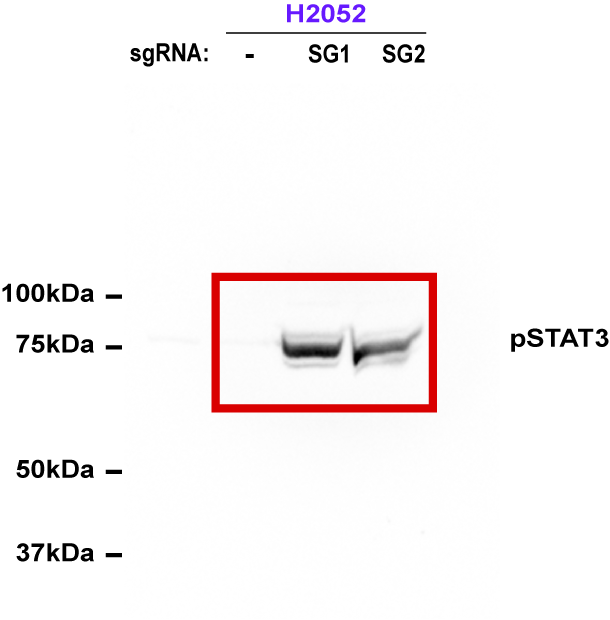

Supplement: Supplementary file 9 — Source data Fig. 3 [file 44319_2024_217_MOESM9_ESM.zip › Figure 3/3E/western H2052 pSTAT3.tif]

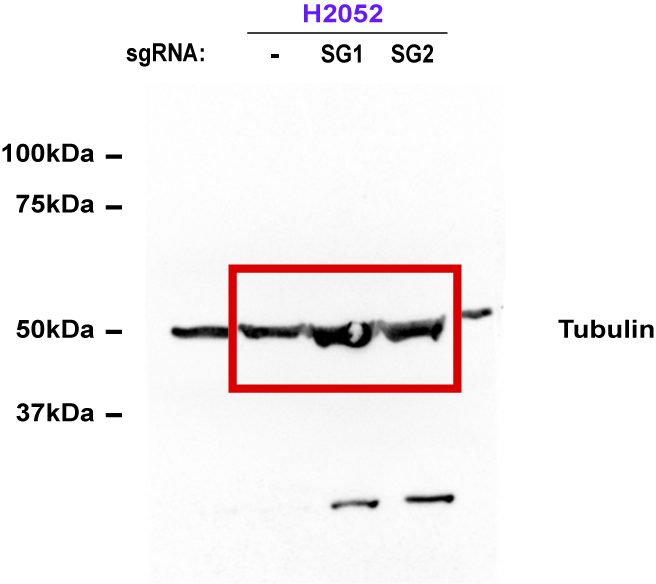

Supplement: Supplementary file 9 — Source data Fig. 3 [file 44319_2024_217_MOESM9_ESM.zip › Figure 3/3E/western H2052 Tubulin.tif]

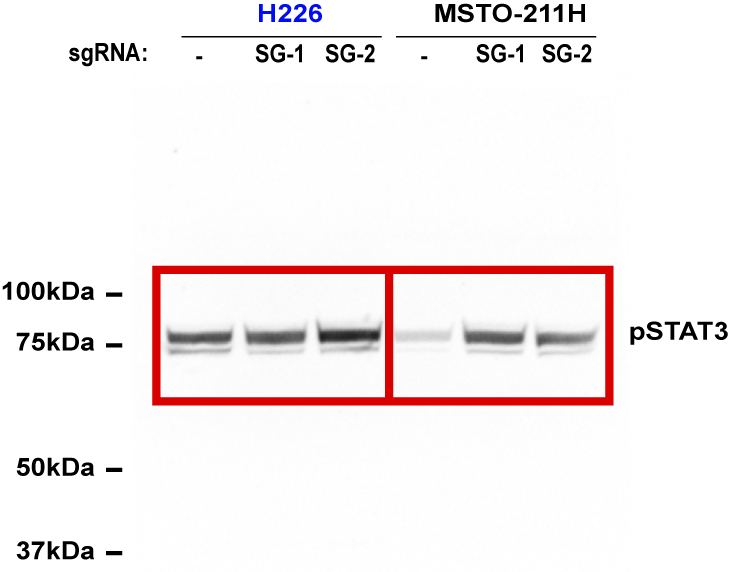

Supplement: Supplementary file 9 — Source data Fig. 3 [file 44319_2024_217_MOESM9_ESM.zip › Figure 3/3E/western H226 211H pSTAT3.tif]

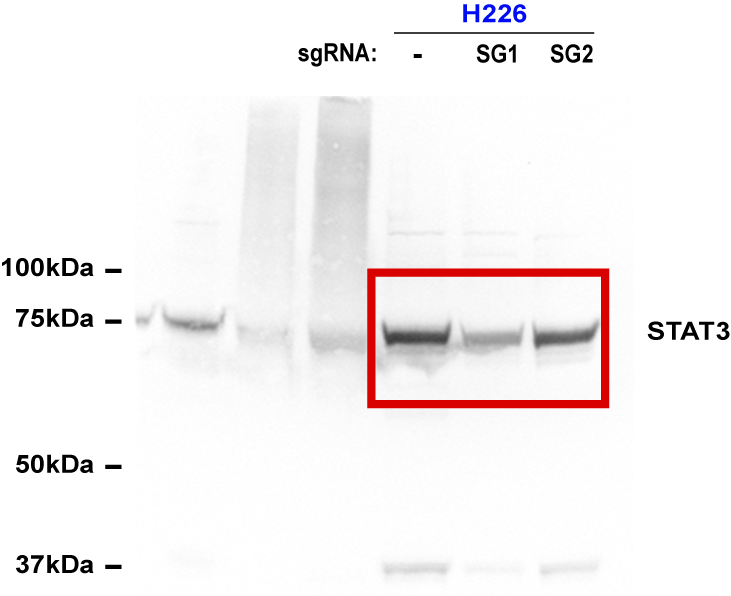

Supplement: Supplementary file 9 — Source data Fig. 3 [file 44319_2024_217_MOESM9_ESM.zip › Figure 3/3E/western H226 STAT3.tif]

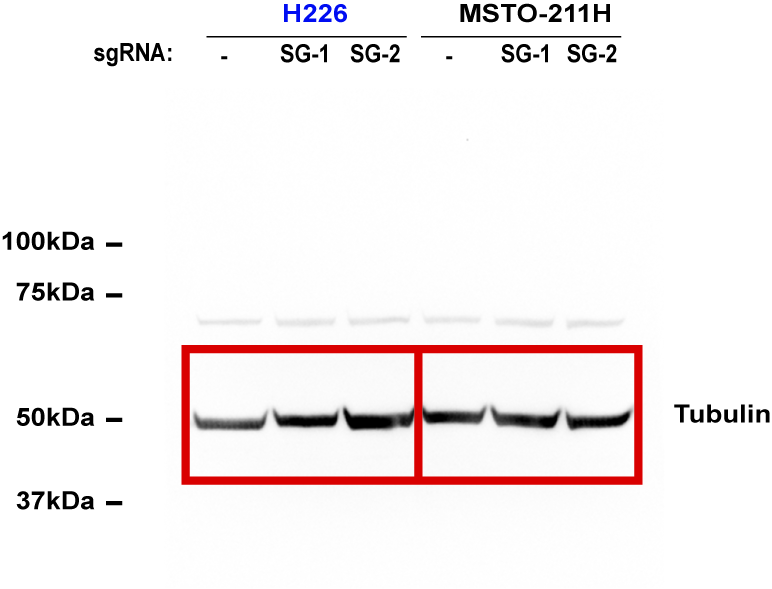

Supplement: Supplementary file 9 — Source data Fig. 3 [file 44319_2024_217_MOESM9_ESM.zip › Figure 3/3E/western H226 211H Tubulin.tif]

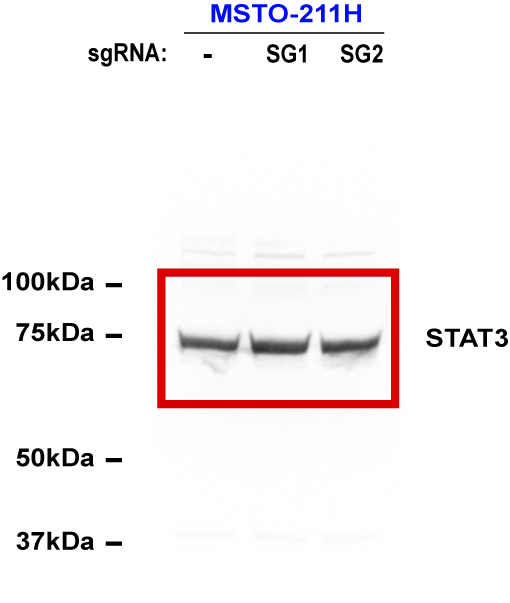

Supplement: Supplementary file 9 — Source data Fig. 3 [file 44319_2024_217_MOESM9_ESM.zip › Figure 3/3E/western 211H STAT3.tif]

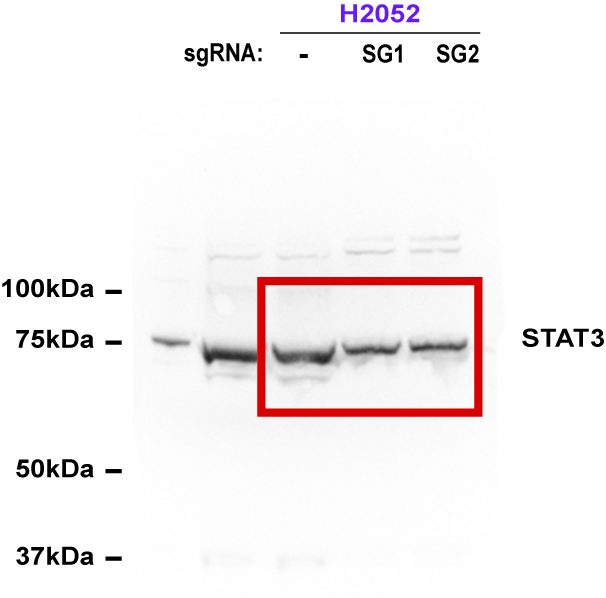

Supplement: Supplementary file 9 — Source data Fig. 3 [file 44319_2024_217_MOESM9_ESM.zip › Figure 3/3E/western H2052 STAT3.tif]

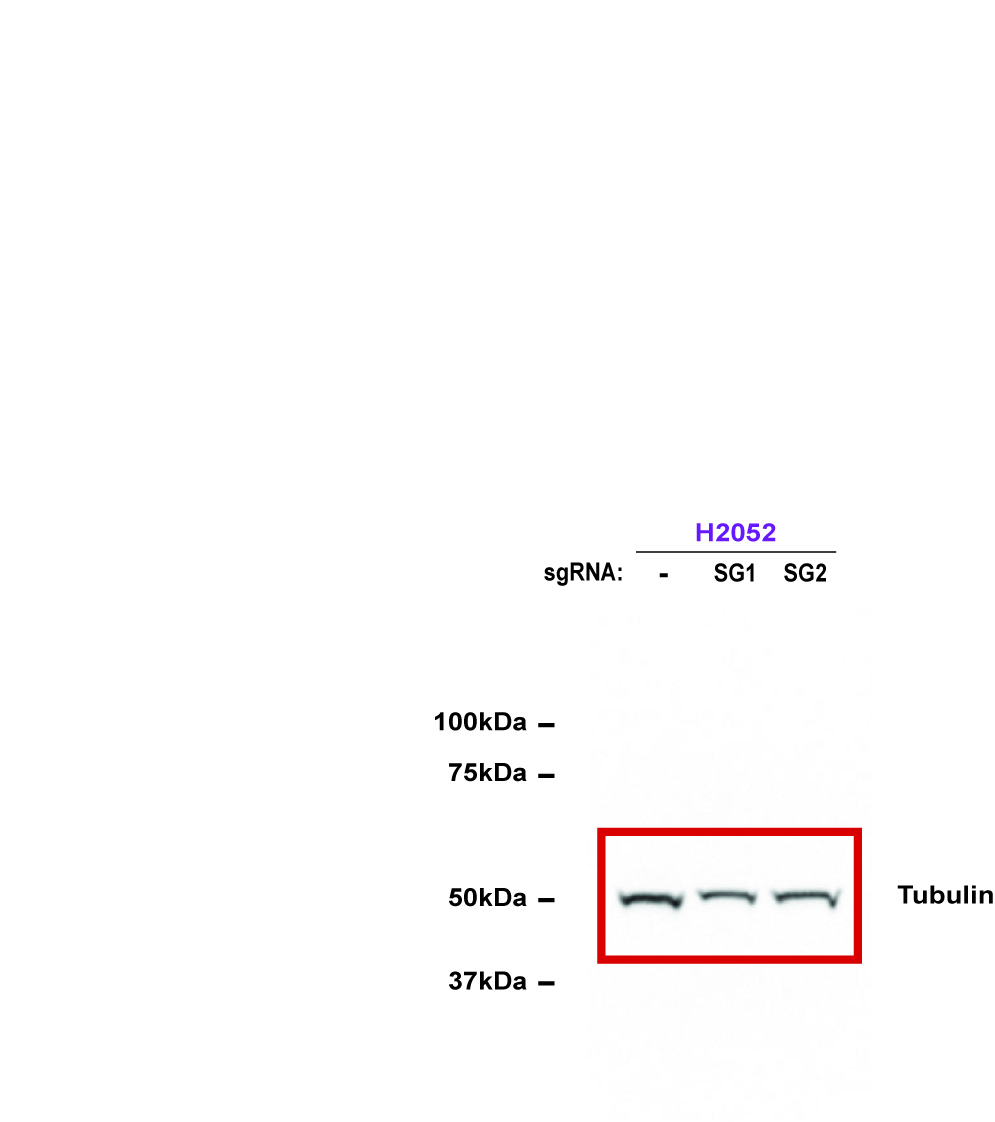

Supplement: Supplementary file 9 — Source data Fig. 3 [file 44319_2024_217_MOESM9_ESM.zip › Figure 3/3D/western H2052 Tubulin.tif]

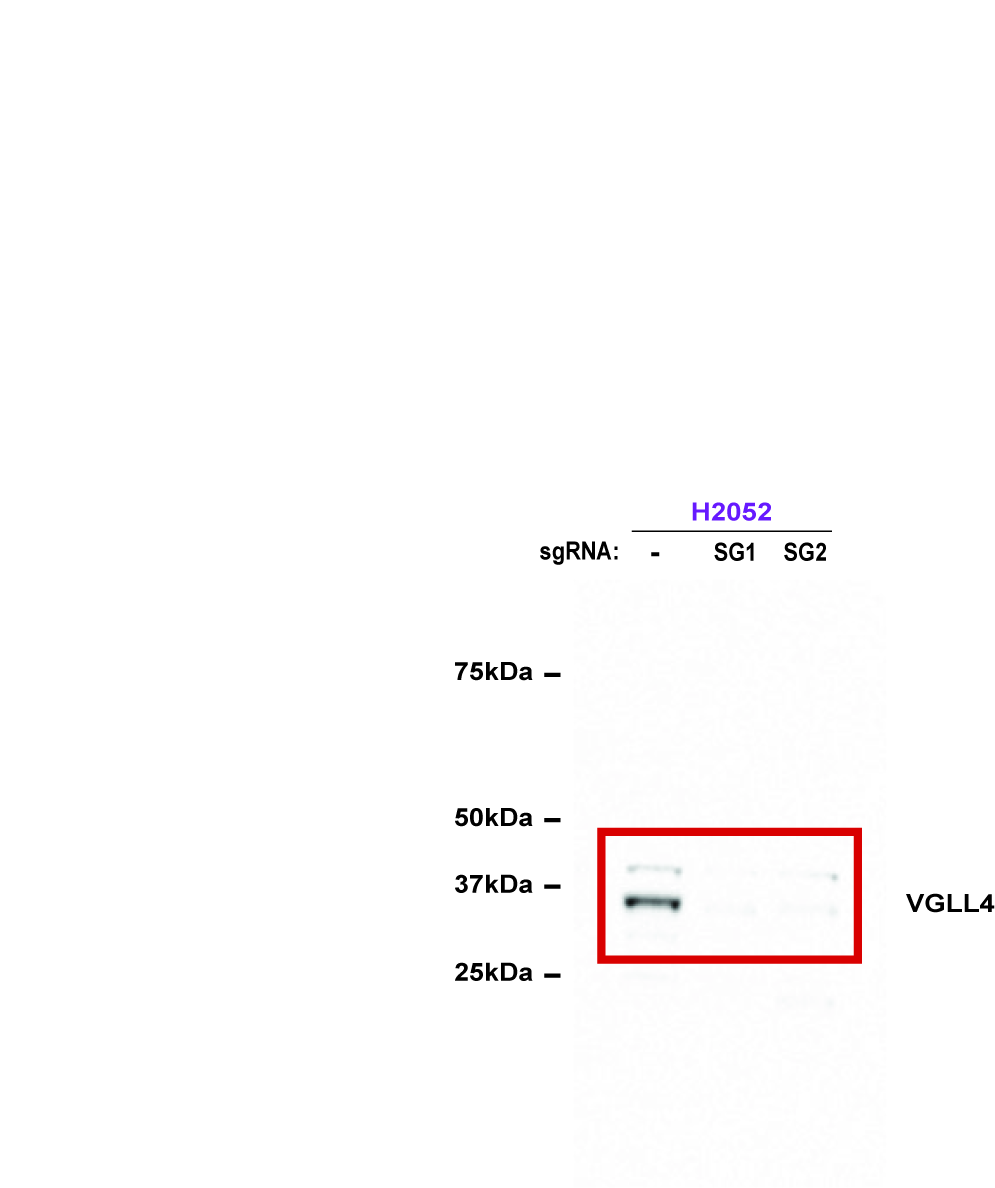

Supplement: Supplementary file 9 — Source data Fig. 3 [file 44319_2024_217_MOESM9_ESM.zip › Figure 3/3D/western H2052 VGLL4.tif]

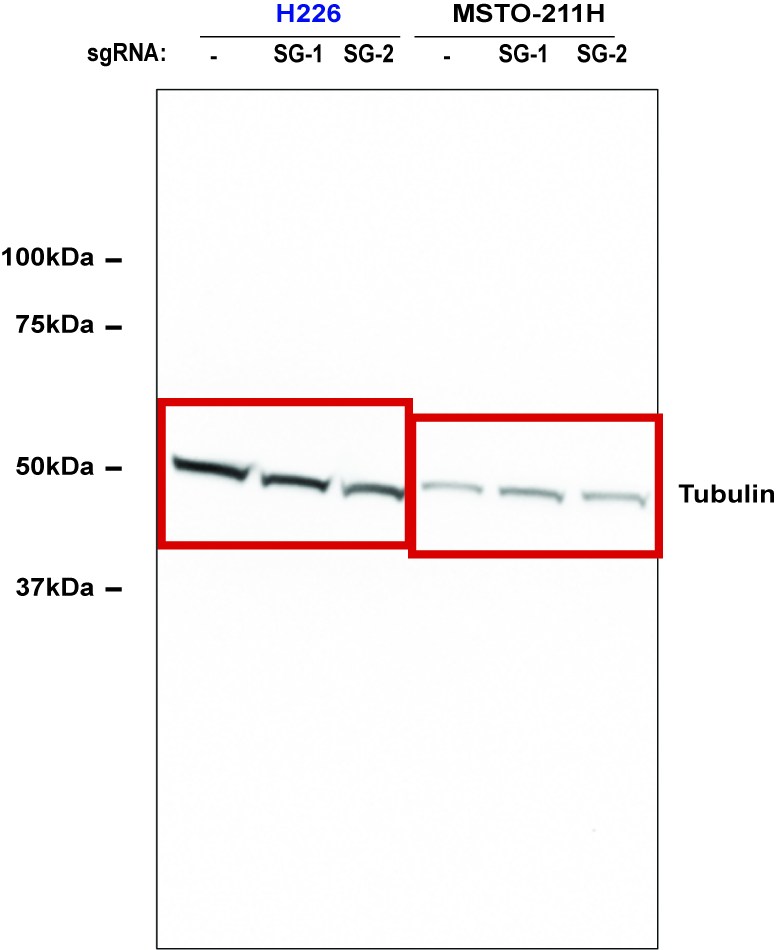

Supplement: Supplementary file 9 — Source data Fig. 3 [file 44319_2024_217_MOESM9_ESM.zip › Figure 3/3D/western H226 211H Tubulin.tif]

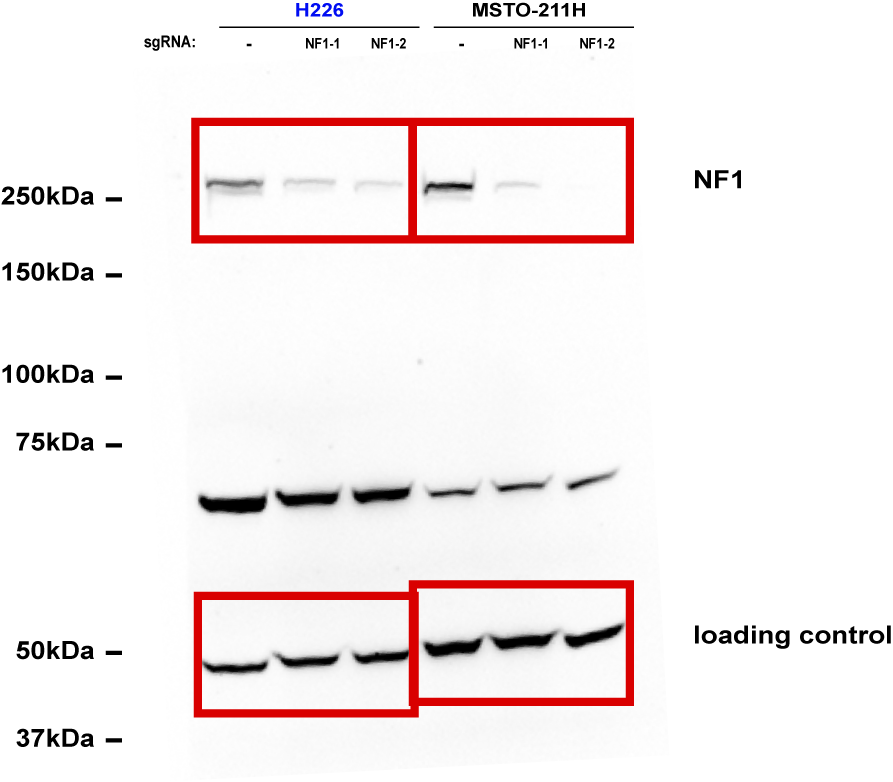

Supplement: Supplementary file 9 — Source data Fig. 3 [file 44319_2024_217_MOESM9_ESM.zip › Figure 3/3D/western H226 211H NF1 and loading control.tif]

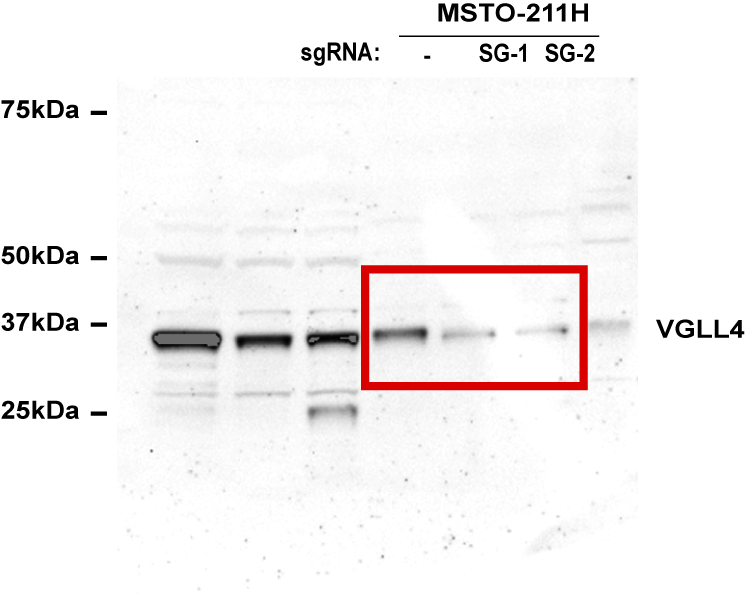

Supplement: Supplementary file 9 — Source data Fig. 3 [file 44319_2024_217_MOESM9_ESM.zip › Figure 3/3D/western 211H VGLL4.tif]

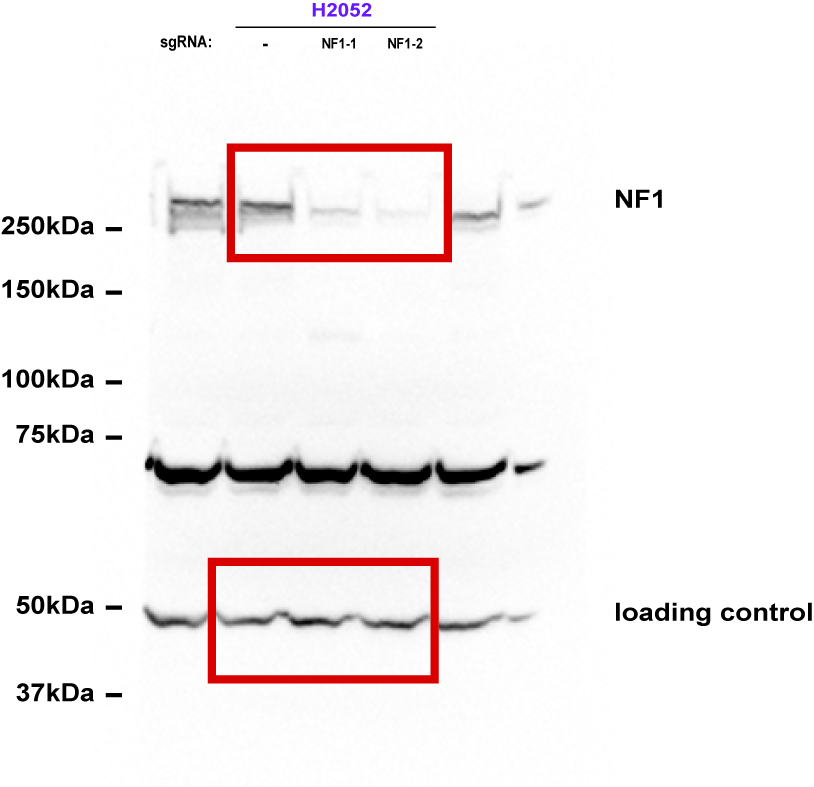

Supplement: Supplementary file 9 — Source data Fig. 3 [file 44319_2024_217_MOESM9_ESM.zip › Figure 3/3D/western H2052 NF1 and loading control.tif]

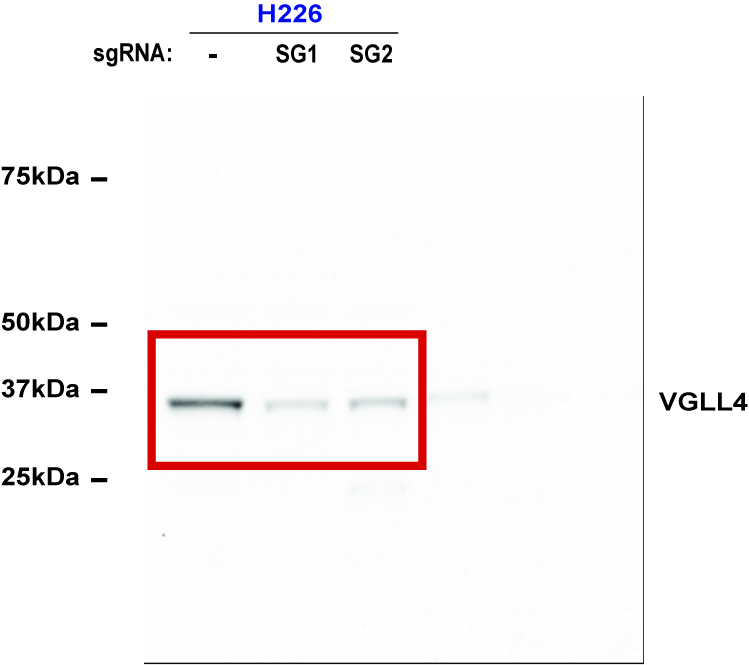

Supplement: Supplementary file 9 — Source data Fig. 3 [file 44319_2024_217_MOESM9_ESM.zip › Figure 3/3D/western H226 VGLL4.tif]

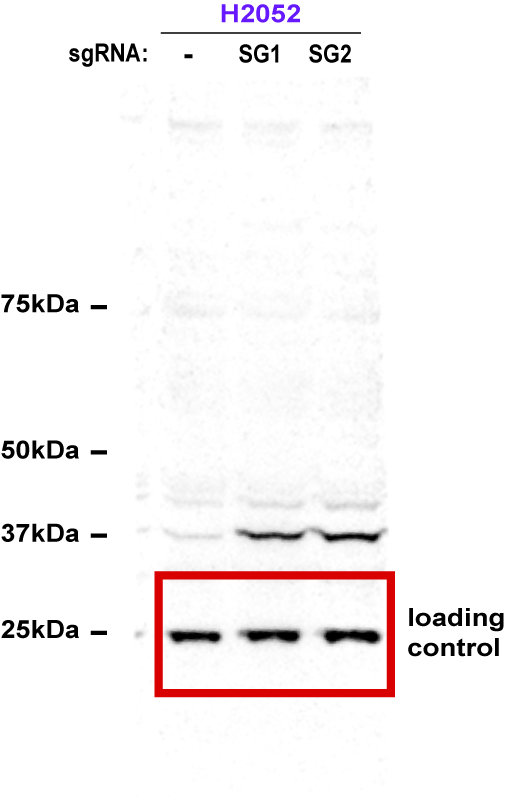

Supplement: Supplementary file 9 — Source data Fig. 3 [file 44319_2024_217_MOESM9_ESM.zip › Figure 3/3F/western H2052 loading control.tif]

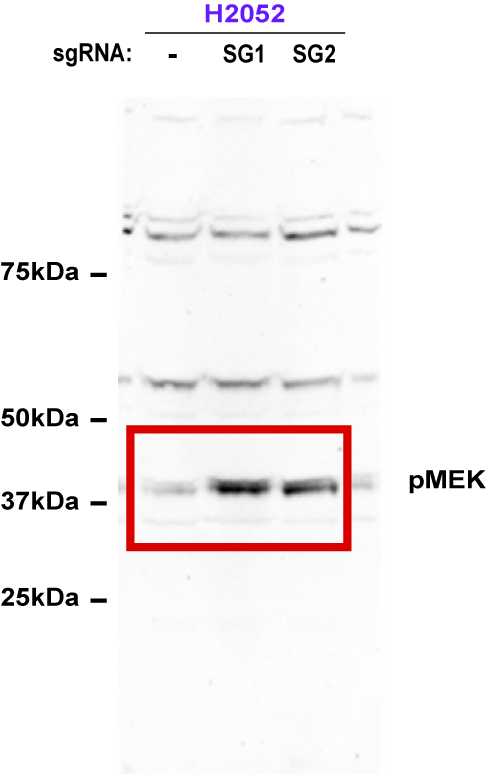

Supplement: Supplementary file 9 — Source data Fig. 3 [file 44319_2024_217_MOESM9_ESM.zip › Figure 3/3F/western H2052 pMEK.tif]

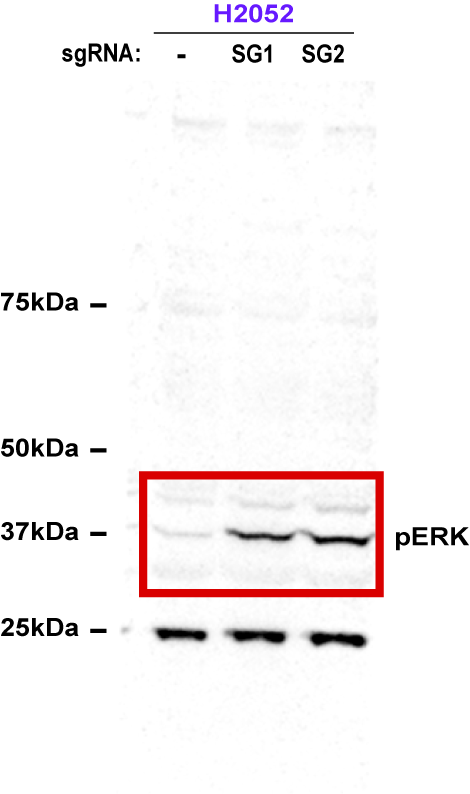

Supplement: Supplementary file 9 — Source data Fig. 3 [file 44319_2024_217_MOESM9_ESM.zip › Figure 3/3F/western H2052 pERK.tif]

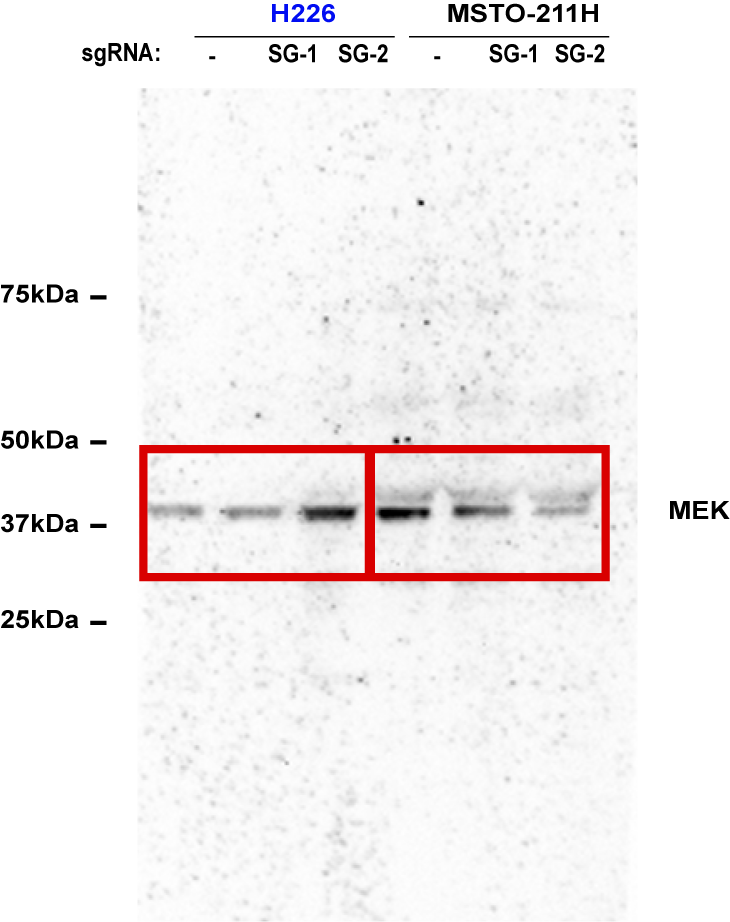

Supplement: Supplementary file 9 — Source data Fig. 3 [file 44319_2024_217_MOESM9_ESM.zip › Figure 3/3F/western H226 211H MEK.tif]

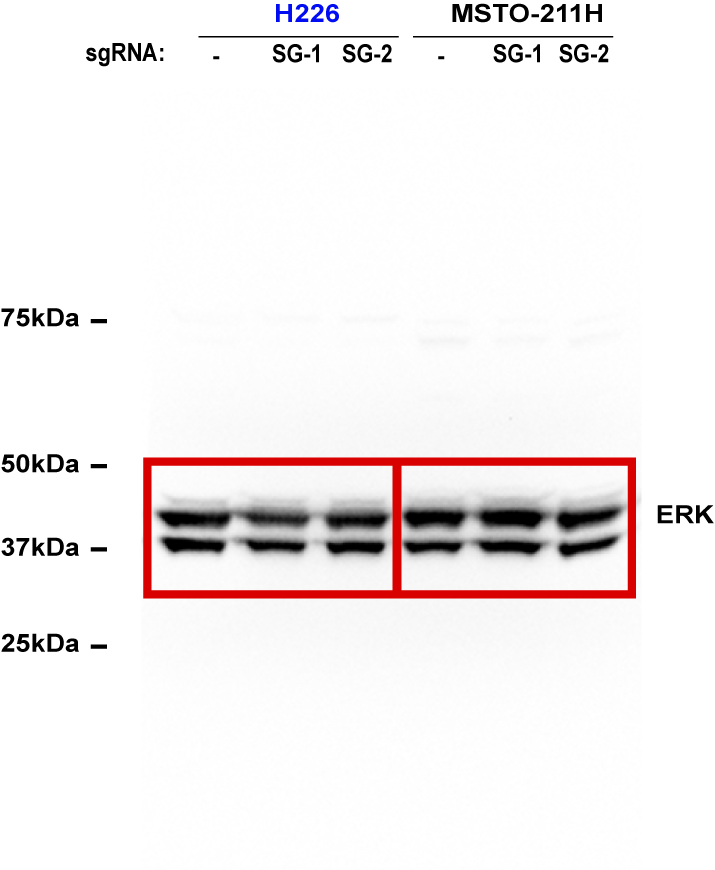

Supplement: Supplementary file 9 — Source data Fig. 3 [file 44319_2024_217_MOESM9_ESM.zip › Figure 3/3F/western H226 211H ERK.tif]

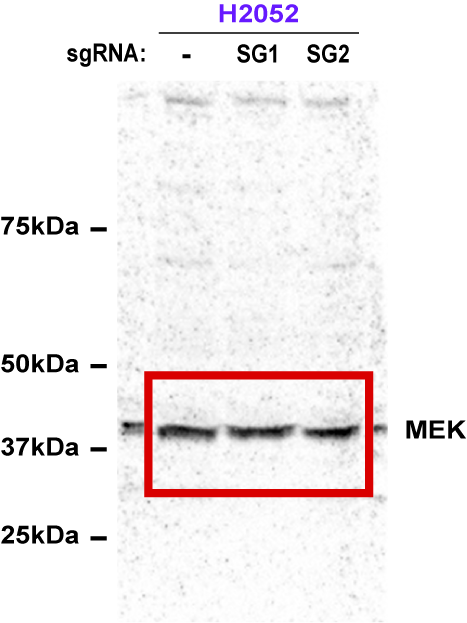

Supplement: Supplementary file 9 — Source data Fig. 3 [file 44319_2024_217_MOESM9_ESM.zip › Figure 3/3F/western H2052 MEK.tif]

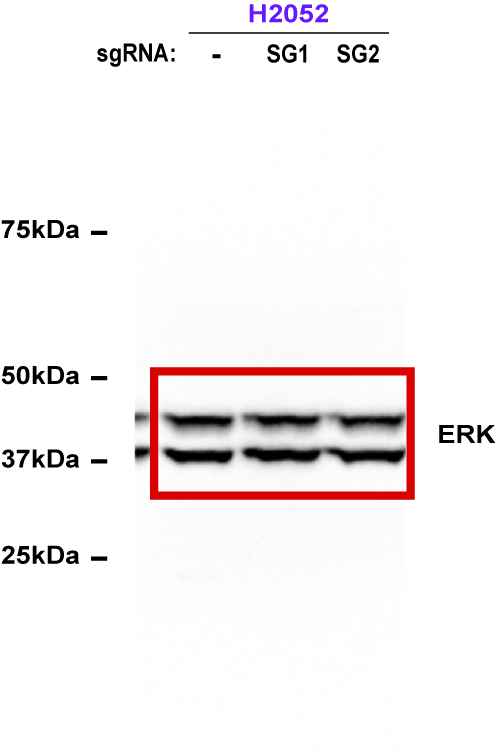

Supplement: Supplementary file 9 — Source data Fig. 3 [file 44319_2024_217_MOESM9_ESM.zip › Figure 3/3F/western H2052 ERK.tif]

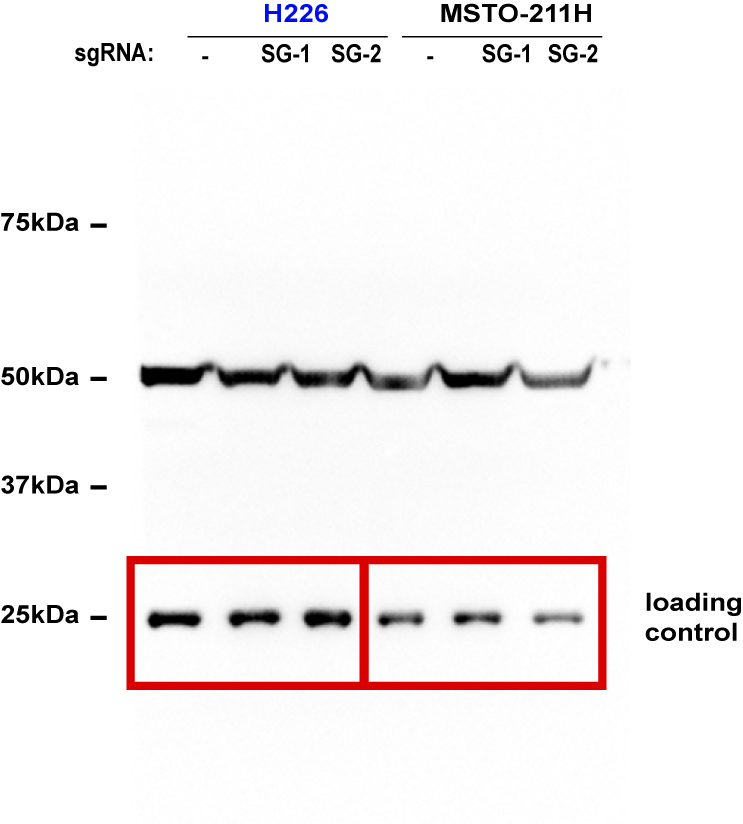

Supplement: Supplementary file 9 — Source data Fig. 3 [file 44319_2024_217_MOESM9_ESM.zip › Figure 3/3F/western H226 211H loading control.tif]

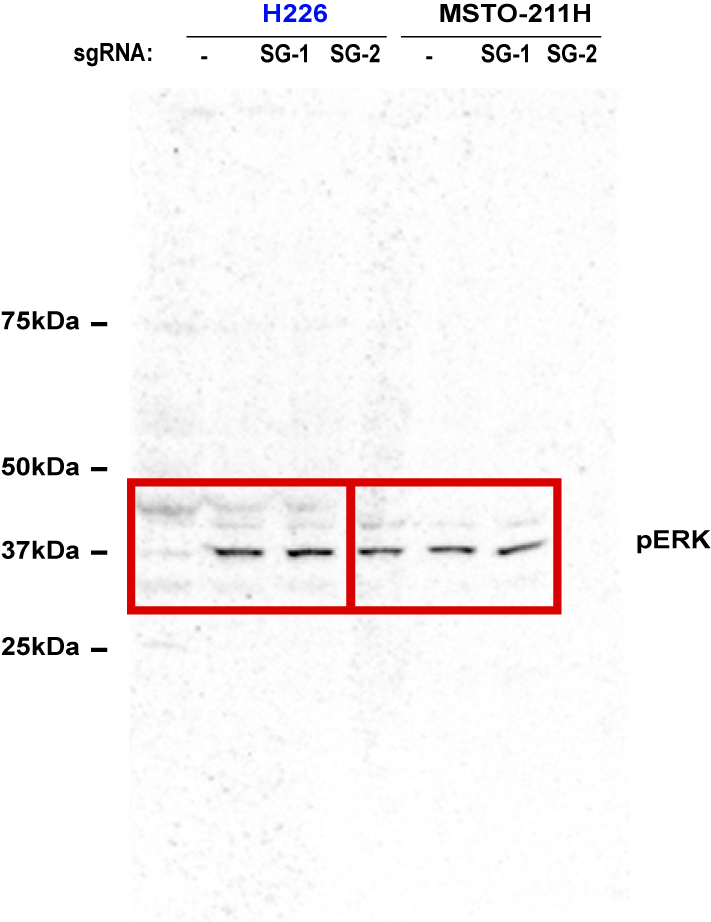

Supplement: Supplementary file 9 — Source data Fig. 3 [file 44319_2024_217_MOESM9_ESM.zip › Figure 3/3F/western H226 211H pERK.tif]

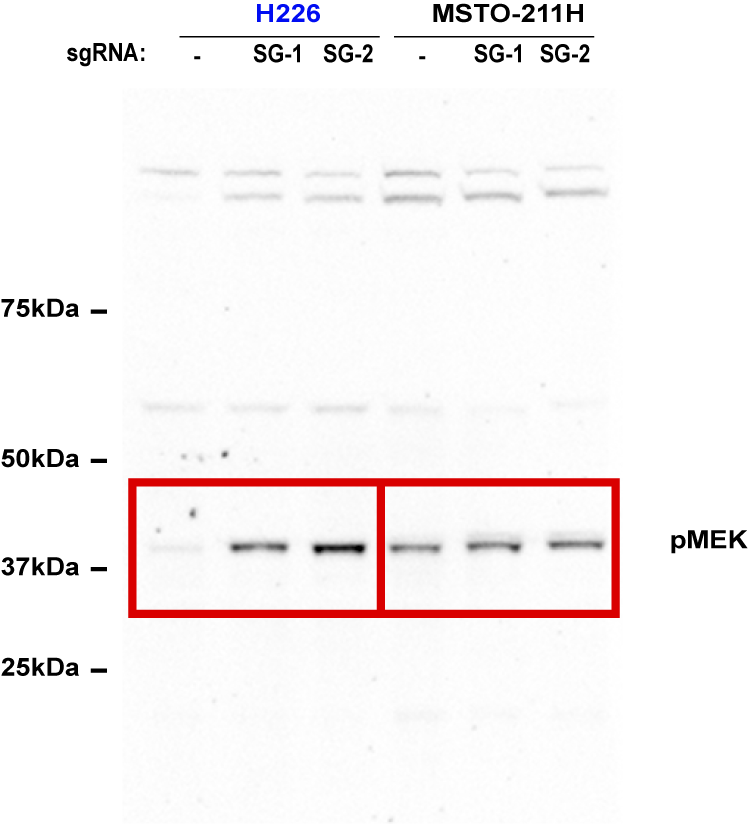

Supplement: Supplementary file 9 — Source data Fig. 3 [file 44319_2024_217_MOESM9_ESM.zip › Figure 3/3F/western H226 211H pMEK.tif]
