# Supplementary material for: Identification of resistance mechanisms to small-molecule inhibition of TEAD-regulated transcription
Source: EMBO Rep. 2024 Aug 5;25(9):14. doi: 10.1038/s44319-024-00217-3 (PMC11387499; doi:10.1038/s44319-024-00217-3)
Supplement: Supplementary file 11 — Expanded View Figures [file 44319_2024_217_MOESM11_ESM.pdf]

# Expanded View Figures

**Figure EV1. The effect of TEAD inhibitors on the transcriptome and proteome of Hippo pathway mutant mesothelioma cells.**

(**A**) Charts of H2052 and H226 cell viability following treatment with different doses of VT107 for 4 days. $n = 3$ biological replicates. (**B**) Multi-dimensional scaling analysis plots of transcriptomes of biological replicates of RNA-seq analyses of H2052 and H226 cells treated with VT107 or DMSO. $n = 3$ biological replicates. (**C**) Gene set enrichment analysis plots of the Cordenonsi YAP signature in differentially expressed genes (VT107 vs DMSO) in H2052 cells and H226 cells. (**D**) Correlation plots comparing the transcriptomes of VT107-treated H2052 and H226 cells. The whole transcriptome is plotted in the left panel and significantly differentially expressed genes only in the right panel. Correlation was assessed using the Pearson's correlation coefficient. (**E**) A multi-dimensional scaling analysis plot of biological replicates of proteomes from H2052 cells treated with VT107 (TEADi) for 1, 4, or 24-h or with DMSO. $n = 5$ biological replicates. (**F**) Volcano plots of protein expression of H2052 cells following 1-h (1) or 4-h (ii) VT107 treatment, $n = 5$ biological replicates for each. Proteins whose abundance changed upon VT107 treatment are highlighted in red. (**G**) Immunoblots of lysates from the indicated cell lines, treated with 3 µM of VT107 or VT108 for 24 h and probed with the specified antibodies. Molecular mass markers are indicated. Data information: In (**A**), data are presented as mean ± SEM.

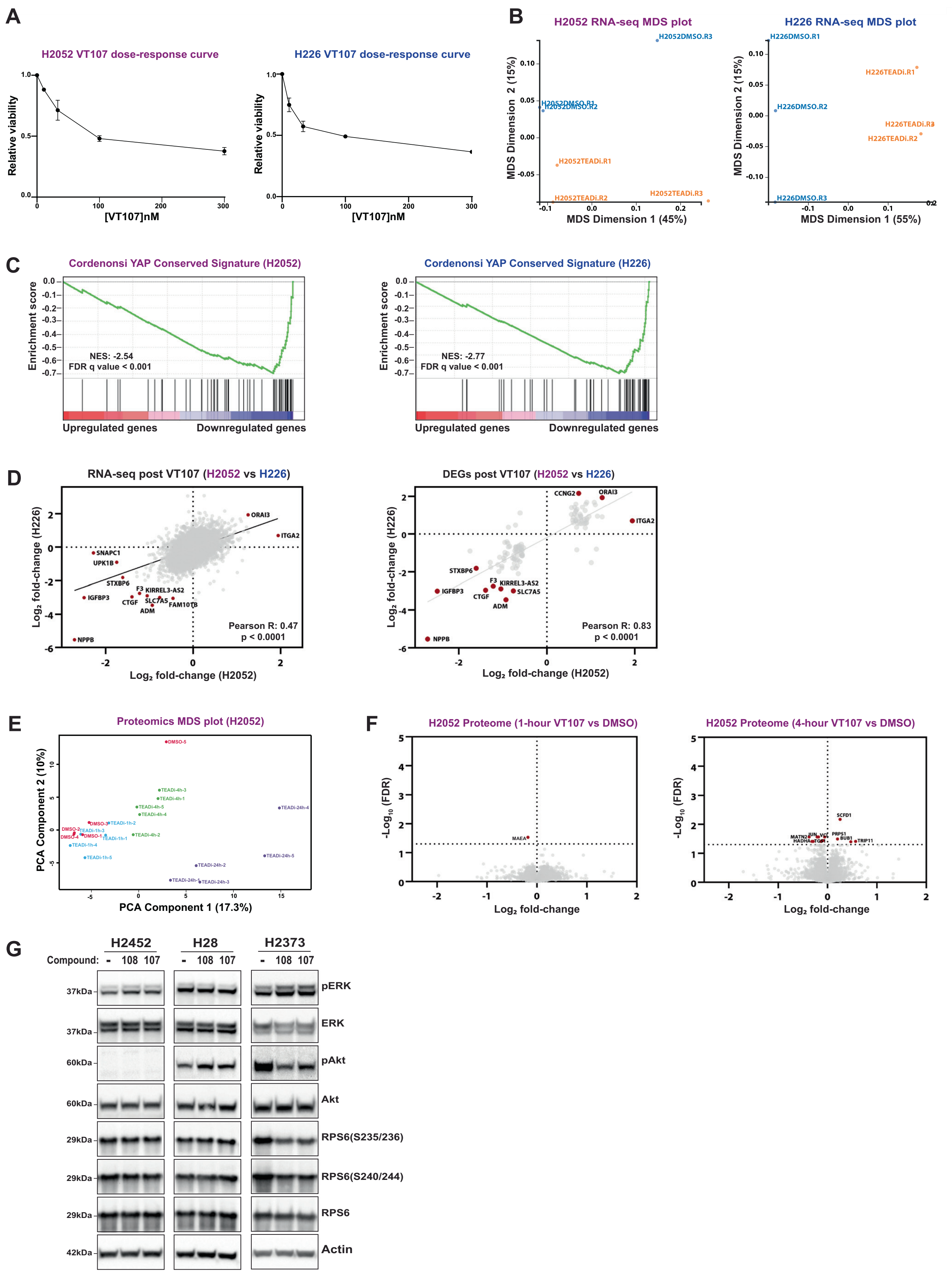
A
H2052 VT107 dose-response curve
H226 VT107 dose-response curve
Relative viability
[VT107]nM
B
H2052 RNA-seq MDS plot
H226 RNA-seq MDS plot
MDS Dimension 2 (15%)
MDS Dimension 1 (45%)
MDS Dimension 1 (55%)
C
Cordenonsi YAP Conserved Signature (H2052)
Cordenonsi YAP Conserved Signature (H226)
Enrichment score
NES: -2.54
FDR q value < 0.001
NES: -2.77
FDR q value < 0.001
Upregulated genes
Downregulated genes
D
RNA-seq post VT107 (H2052 vs H226)
DEGs post VT107 (H2052 vs H226)
Log2 fold-change (H226)
Log2 fold-change (H2052)
Pearson R: 0.47
p < 0.0001
Pearson R: 0.83
p < 0.0001
E
Proteomics MDS plot (H2052)
PCA Component 2 (10%)
PCA Component 1 (17.3%)
F
H2052 Proteome (1-hour VT107 vs DMSO)
H2052 Proteome (4-hour VT107 vs DMSO)
-Log10 (FDR)
Log2 fold-change
G
H2452
H28
H2373
Compound: - 108 107
pERK
ERK
pAkt
Akt
RPS6(S235/236)
RPS6(S240/244)
RPS6
Actin

 © The Author(s)

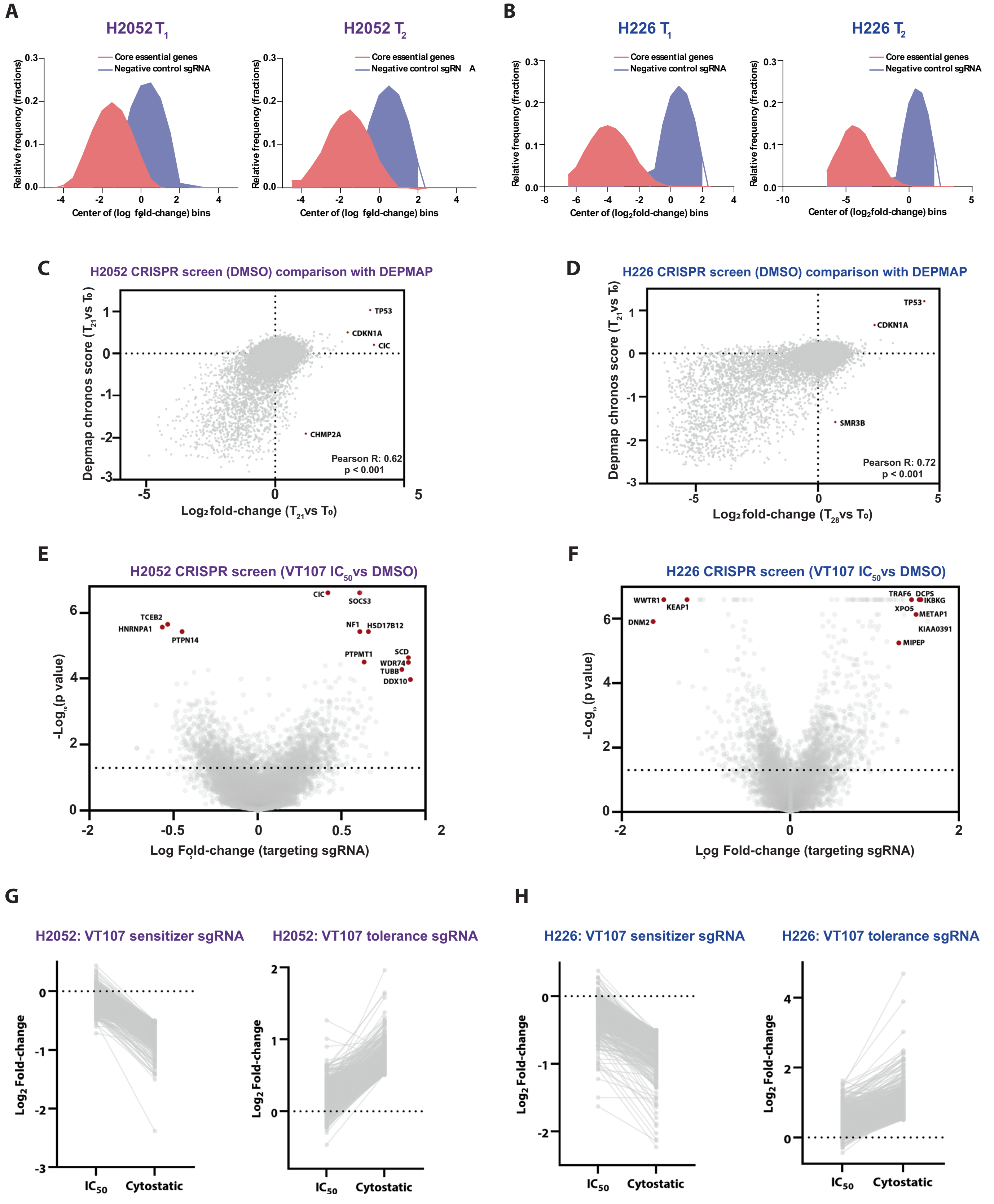
A
H2052 T1
H2052 T2
Core essential genes
Negative control sgRNA
Relative frequency (fractions)
Center of (log2 fold-change) bins
B
H226 T1
H226 T2
C
H2052 CRISPR screen (DMSO) comparison with DEPMAP
TP53
CDKN1A
CIC
CHMP2A
Pearson R: 0.62
p < 0.001
Depmap chronos score (T21 vs T0)
Log2 fold-change (T21 vs T0)
D
H226 CRISPR screen (DMSO) comparison with DEPMAP
SMR3B
Pearson R: 0.72
Log2 fold-change (T28 vs T0)
E
H2052 CRISPR screen (VT107 IC50 vs DMSO)
SOCS3
TCEB2
HNRNPA1
PTPN14
NF1
HSD17B12
PTPMT1
SCD
WDR74
TUBB
DDX10
-Log10 (p value)
Log2 Fold-change (targeting sgRNA)
F
H226 CRISPR screen (VT107 IC50 vs DMSO)
WWTR1
KEAP1
DNM2
TRAF6
DCPS
IKBKG
XPO5
METAP1
KIAA0391
MIPEP
G
H2052: VT107 sensitizer sgRNA
H2052: VT107 tolerance sgRNA
Log2 Fold-change
IC50
Cytostatic
H
H226: VT107 sensitizer sgRNA
H226: VT107 tolerance sgRNA

**Figure EV2. TEAD inhibitor CRISPR/Cas9 screens in mesothelioma cell lines.**

(**A**, **B**) Histograms representing the quantity of sgRNAs targeting core essential genes, or non-targeting control sgRNAs from the Brunello library, within the DMSO-treatment populations at the endpoint of $IC_{50}$ or cytostatic dose treatment with VT107 of H2052 cells (**A**) and H226 cells (**B**). (**C**, **D**) Correlation plots comparing data from our genome-wide CRISPR/Cas9 screens in DMSO-treated H2052 cells (**C**) or H226 cells (**D**) and data from the Depmap database. Correlation was assessed using the Pearson's correlation coefficient. (**E**, **F**) Volcano plots representing the overall change of targeting sgRNAs (per gene) in response to VT107 at the $IC_{50}$ dose during the CRISPR/Cas9 screens from H2052 cells ($IC_{50}$: 18 nM) (**E**) or H226 cells ($IC_{50}$: 33 nM) (**F**). (**G**, **H**) Comparison of the $\log_2$ fold-change of targeting gRNAs that conferred VT107 sensitivity or resistance at the $IC_{50}$ and cytostatic VT107 doses in H2052 cells (**G**) or H226 cells (**H**) during the CRISPR/Cas9 screens. Data information: In (**E**, **F**), significance was assessed using empirical Bayesian statistics.

 © The Author(s)

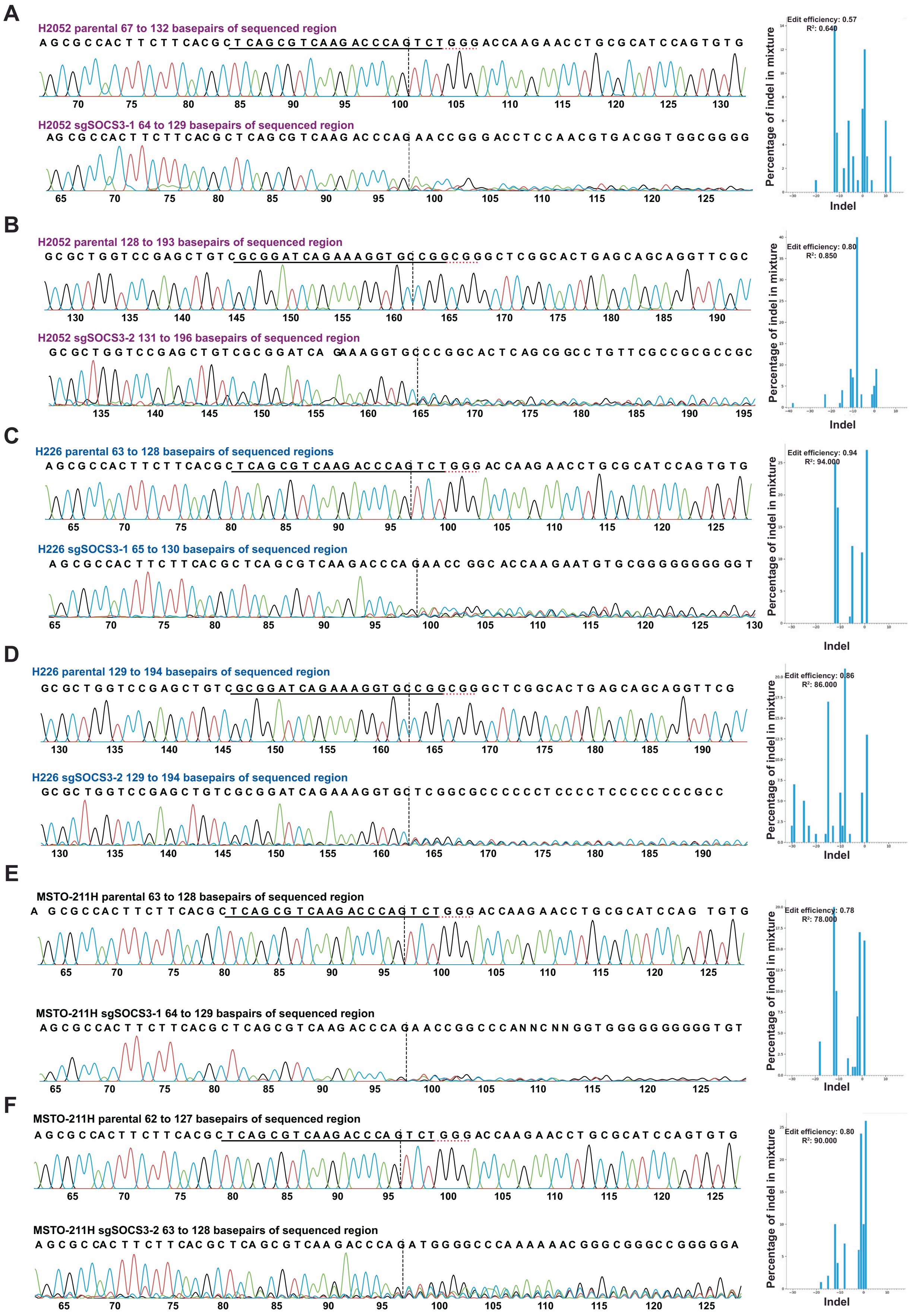
A
H2052 parental 67 to 132 basepairs of sequenced region
H2052 sgSOCS3-1 64 to 129 basepairs of sequenced region
Edit efficiency: 0.57
R²: 0.640
B
H2052 parental 128 to 193 basepairs of sequenced region
H2052 sgSOCS3-2 131 to 196 basepairs of sequenced region
Edit efficiency: 0.80
R²: 0.850
C
H226 parental 63 to 128 basepairs of sequenced regions
H226 sgSOCS3-1 65 to 130 basepairs of sequenced region
Edit efficiency: 0.94
R²: 94.000
D
H226 parental 129 to 194 basepairs of sequenced region
H226 sgSOCS3-2 129 to 194 basepairs of sequenced region
Edit efficiency: 0.86
R²: 86.000
E
MSTO-211H parental 63 to 128 basepairs of sequenced region
MSTO-211H sgSOCS3-1 64 to 129 baspairs of sequenced region
Edit efficiency: 0.78
R²: 78.000
F
MSTO-211H parental 62 to 127 basepairs of sequenced region
MSTO-211H sgSOCS3-2 63 to 128 basepairs of sequenced region
Edit efficiency: 0.80
R²: 90.000
Percentage of indel in mixture
Indel

**Figure EV3. CRISPR/Cas9-induced mutagenesis of *SOCS3* in H2052, H226, and MSTO-211H cells.**

(**A–F**) DNA sequence alignment charts at independent sgRNA-targeted regions of the *SOCS3* gene in parental or sgRNA-expressing H2052 (**A**, **B**), H226 (**C**, **D**) and MSTO-211H (**E**, **F**) cells. Corresponding sgRNA and PAM sequences are underlined in black and red, respectively. TIDE-analysis calculations of CRISPR/Cas9-induced mutagenesis efficiency are indicated in bar charts on the right.

 © The Author(s)

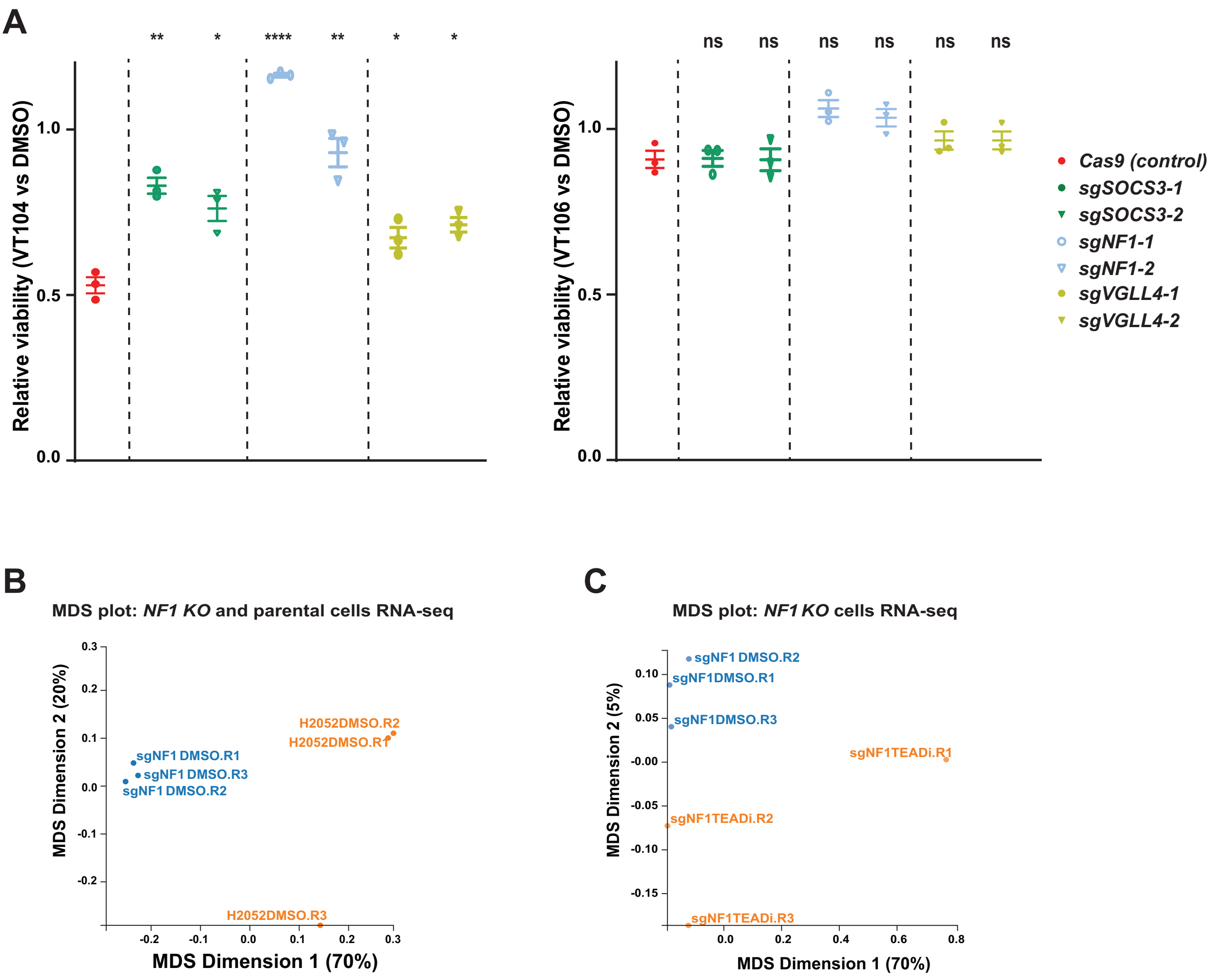


**Figure EV4. Mutation of *NF1, SOCS3,* or *VGLL4* in NCI-H2052 cells induces resistance to the VT104 TEAD inhibitor.**

(**A**) Charts of the impact of VT104 or VT106 on the viability of parental H2052 cells and *NF1, SOCS3,* or *VGLL4* mutant H2052 cells, as assessed by alamar blue assays. Cells were treated with 100 nM VT104 or VT106 for 4 days. $n = 3$ biological replicates. (**B**, **C**) Multi-dimensional scaling analysis plots comparing biological replicates of RNA-seq experiments performed on parental and *NF1* mutant H2052 cells treated with either DMSO or VT107 for 24 h. $n = 3$ biological replicates. Data information: In (**A**), data are presented as mean ± SEM. $*p < 0.05$, $**p < 0.01$, $****p < 0.0001$, ns—not significant. (Student's t-tests). *P* values for VT104 chart were (from left to right): 0.00329; 0.03053; 0.00004; 0.00178; 0.03383; 0.01008. *P* values for VT106 chart were (from left to right): 0.89364; 0.95685; 0.0735; 0.15201; 0.48852; 0.48270.

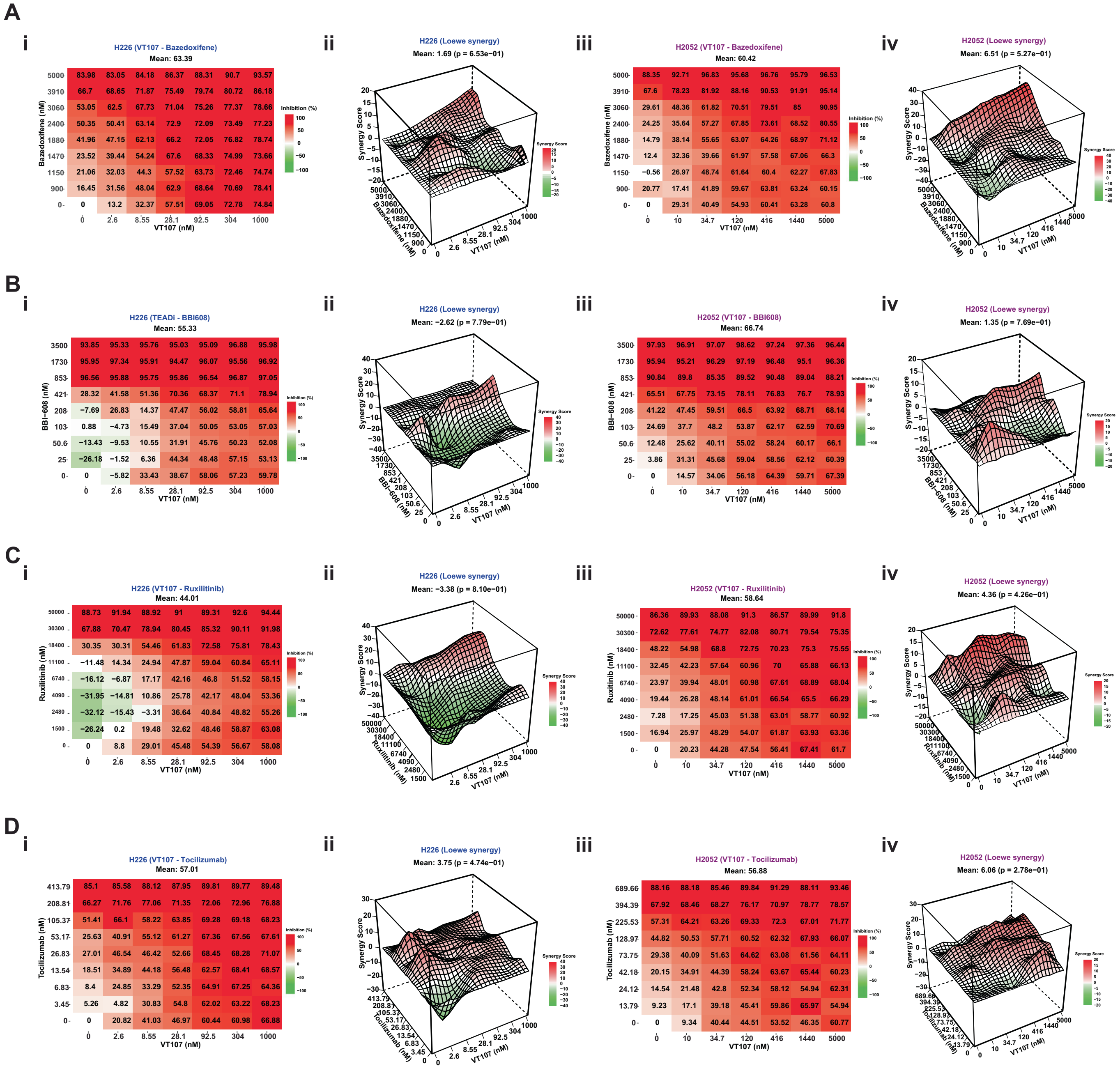

**Figure EV5. The impact of combined JAK/STAT pathway inhibitors and TEAD inhibitors on mesothelioma cells.**

(**A–D**) Dose-response matrices indicating the level of inhibition of cell number by treatment with different doses of VT107 and/or different JAK/STAT pathway inhibitors in H2052 cells (i) and H226 cells (iii). 3D synergy calculation topological maps indicating the type and degree of interaction between VT107 and different JAK/STAT pathway inhibitors in H2052 cell numbers (ii) and H226 cell numbers (iv). JAK/STAT pathway inhibitors that were used were Bazedoxifene (**A**), BBI-608 (**B**), Ruxilitinib (**C**) and Tocilizumab (**D**). In all assays, $n = 2$ biological replicates (2 technical replicates were performed for each biological replicate). Mean synergy scores were tested for statistical significance using the parametric bootstrapping method, $n = 2$ biological replicates.

 © The Author(s)

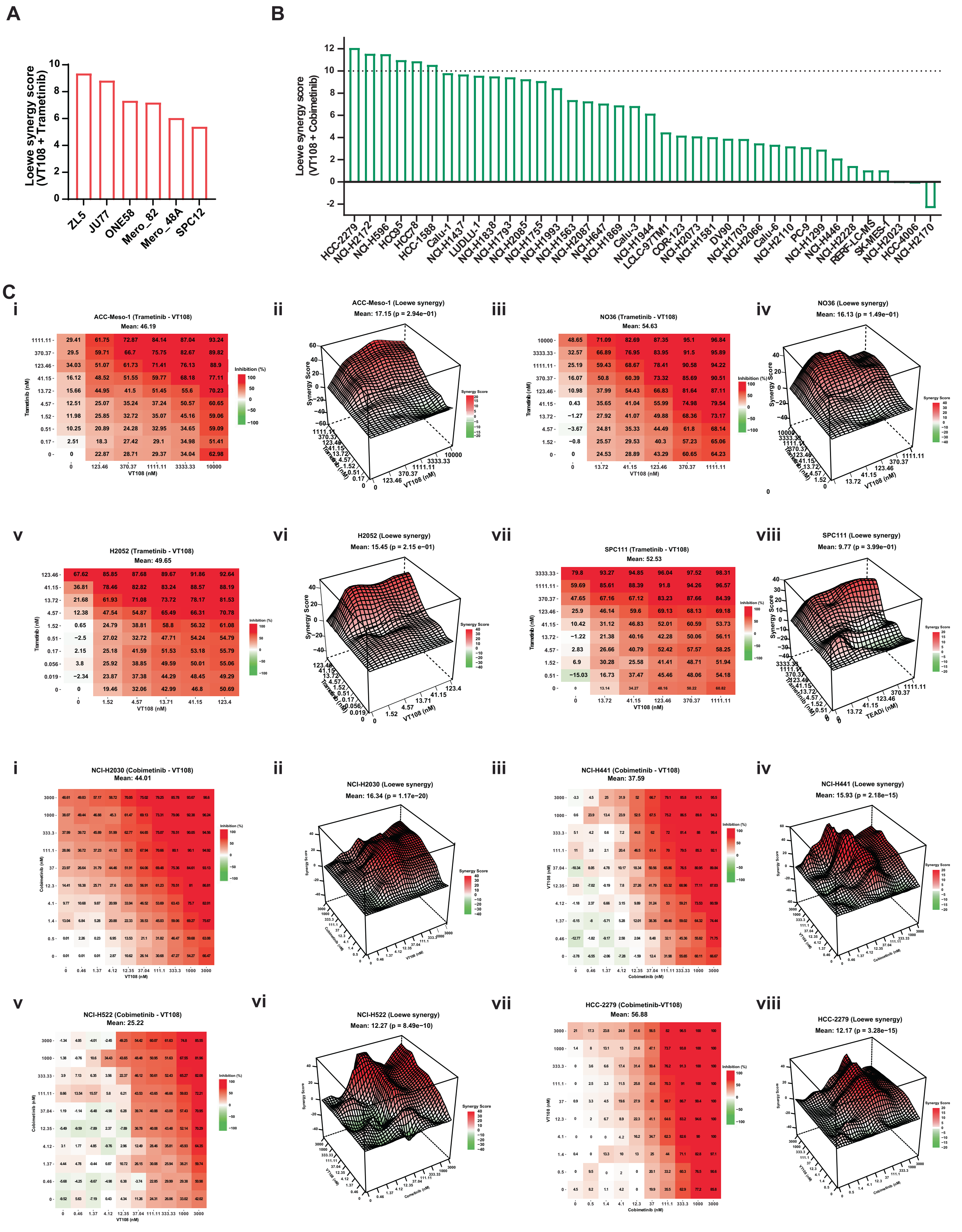
A
Loewe synergy score (VT108 + Trametinib)
ZL5
JU77
ONE58
Mero_82
Mero_48A
SPC12
B
Loewe synergy score (VT108 + Cobimetinib)
HCC-2279
NCI-H2172
NCI-H596
HCC95
HCC78
HCC-1588
Calu-1
NCI-H1437
LUDLU-1
NCI-H1838
NCI-H1793
NCI-H2085
NCI-H1755
NCI-H1993
NCI-H1563
NCI-H2087
NCI-H647
NCI-H1869
Calu-3
NCI-H1944
LCLC-97TM1
COR-123
NCI-H2073
NCI-H1581
DV90
NCI-H1703
NCI-H2066
Calu-6
NCI-H2110
PC9
NCI-H1299
NCI-H446
NCI-H2228
REFF-LC-MS
SK-MES-1
NCI-H2023
HCC4006
NCI-H2170
C
i ACC-Meso-1 (Trametinib - VT108) Mean: 46.19
ii ACC-Meso-1 (Loewe synergy) Mean: 17.15 (p = 2.94e−01)
iii NO36 (Trametinib - VT108) Mean: 54.63
iv NO36 (Loewe synergy) Mean: 16.13 (p = 1.49e−01)
v H2052 (Trametinib - VT108) Mean: 49.65
vi H2052 (Loewe synergy) Mean: 15.45 (p = 2.15 e−01)
vii SPC111 (Trametinib - VT108) Mean: 52.53
viii SPC111 (Loewe synergy) Mean: 9.77 (p = 3.99e−01)
i NCI-H2030 (Cobimetinib - VT108) Mean: 44.01
ii NCI-H2030 (Loewe synergy) Mean: 16.34 (p = 1.17e−20)
iii NCI-H441 (Cobimetinib - VT108) Mean: 37.59
iv NCI-H441 (Loewe synergy) Mean: 15.93 (p = 2.18e−15)
v NCI-H522 (Cobimetinib - VT108) Mean: 25.22
vi NCI-H522 (Loewe synergy) Mean: 12.27 (p = 8.49e−10)
vii HCC-2279 (Cobimetinib-VT108) Mean: 56.88
viii HCC-2279 (Loewe synergy) Mean: 12.17 (p = 3.28e−15)
Inhibition (%)
Synergy Score
Trametinib (nM)
Cobimetinib (nM)
VT108 (nM)
TEADi (nM)

**Figure EV6. The impact of combined MAPK pathway inhibitors and TEAD inhibitors on mesothelioma and NSCLC cells.**

(**A**, **B**) Bar charts indicating the interaction of combining VT108 and MEK inhibitors (trametinib or cobimetinib) on cell numbers of the indicated mesothelioma cell lines (**A**) or NSCLC cell lines (**B**). Synergistic interactions were indicated by synergy scores ≥10 and additive interactions by scores within the range of −10 to 10. $n = 2$ technical replicates. (**C**) Synergistic interactions between TEADi and MEKi in mesothelioma or NSCLC cell lines. Left panels: dose-response matrices indicating the level of inhibition of cell number by treatment of cells with the indicated doses of VT108 and either trametinib or cobimetinib. Right panels: Representative 3D synergy calculation topological maps indicating the type and degree of interaction between drugs on inhibition of cell number. Mean synergy scores were tested for statistical significance using the parametric bootstrapping method, $n = 2$ biological replicates.

 © The Author(s)
